# Predictive Role of Preoperative Whole-Body 18F-FDG PET/CT for Risk Stratification of Early-Stage (FIGO I-IIA) Cervical Cancer Patients Treated by Surgery

**DOI:** 10.7759/cureus.53107

**Published:** 2024-01-28

**Authors:** Nagesh Kumar Singaram, Narendra Hulikal, Ranadheer Manthri, Amith Kumar Chowhan

**Affiliations:** 1 Surgical Oncology, Sri Venkateswara Institute of Medical Sciences, Tirupati, IND; 2 Nuclear Medicine, MNJ Institute of Oncology Regional Cancer Center, Hyderabad, IND; 3 Pathology, All India Institute of Medical Sciences, Raipur, IND

**Keywords:** predictive, suvmax, 18f-fdg-pet/ct, surgery, early-stage cervical cancer

## Abstract

Introduction: The aim of the present study was to investigate the predictive value of maximum standardized uptake value (SUV_max_) measured on preoperative ^18^F-fluoro-2-deoxy-D-glucose positron emission tomography/computed tomography (^18^F-FDG PET/CT) in International Federation of Gynecology and Obstetrics (FIGO 2009) stage I-IIA cervical cancer patients who were treated with radical hysterectomy.

Methods: A total of 47 patients with FIGO stage I-IIA cervical cancer who were evaluated preoperatively with biopsy and ^18^F-FDG PET/CT followed by radical hysterectomy were included in the study. Correlation between SUV_max_ and pathological risk factors or survival was studied.

Results: The mean SUV_max_ was significantly higher in patients with large tumor size (≥4 cm), advanced stage (IIA>IB>IA) and depth of invasion >50%. No significant difference was noted in SUV_max_ between patients with and without pelvic lymph node involvement (P=0.639). SUV_max_ of the primary tumor with and without lymph-vascular invasion were 12.95 and 10.35, respectively (P=0.5). No significant difference was noted between patients with high SUV_max_ and low SUV_max_ with regards to overall survival (OS) and disease-free survival (DFS), using an optimal cut-off value of 7.65 for OS and DFS obtained from receiver operating characteristic (ROC) curve analysis. Patient with tumor size >4cm had 5.9 times more probability of mortality compared to tumor size <4cm (P=0.09).

Conclusion: The present study observations showed that although SUV_max_ is associated with pathological variables, it does not independently predict oncological outcomes in FIGO stage IA-IIA cervical cancer patients who were treated with radical hysterectomy. These findings suggest that SUV_max_ of primary tumor may be used for risk stratification, but not for prognostication in surgically treated early-stage cervical cancer patients. Not using other parameters of ^18^F-FDG PET/CT like metabolic tumor volume (MTV), tumor lysis glycolysis (TLG), small sample size, variation in calculation of SUV_max_, histopathologic heterogeneity, inclusion of stage IA patients in the study were constraints of present study. Further studies with large sample size using multi metabolic parameters of ^18^F-FDG PET/CT, including the SUV_max_,SUV_mean_,SUV_peak_, MTV and TLG are needed.

## Introduction

Carcinoma cervix is one of the most common malignancies in women with an incidence of 16.5% and mortality rate of 7.5% [1]. Patients with locally advanced and early-stage cervical cancer were treated by chemoradiotherapy and surgery respectively [2-5]. Approximately one fourth of early stage (International Federation of Gynecology and Obstetrics (FIGO) I to IIA) patients develop recurrence. Pathological factors like size, histological type, lymph-vascular space invasion (LVSI), and lymph node status have been used to assess the risk of recurrence [6-9]. Postoperative radiotherapy with or without chemotherapy was given to patients with high risk of recurrence [3,4,9,10]. Selection of patients for adjuvant therapy is important because of its effect on survival and quality of life [4,9]. Identification of independent marker that is associated with biological behavior of cervical cancer is needed along with conventional clinicopathological factors for tailoring the treatment and to avoid dual modality treatment, thereby improving the outcomes in early-stage cervical cancer patients. In many cancers, ^18^F-fluoro-2-deoxy-D-glucose positron emission tomography/computed tomography (^18^F-FDG PET/CT) was used for diagnosis, staging and response assessment [11]. In patients with ovarian cancer and endometrial cancer maximum standardized uptake value ( SUV_max_), measured on ^18^F-FDG PET/CT was found to be useful for diagnosis and prognosis [12-15]. ^18^F-FDG PET/CT has high clinical impact in management of gynecological cancers as it can alter the management plan [16]. The role of ^18^F-FDG PET/CT as a staging tool in cervical cancer was confirmed in previous study [17]. Though the association between SUV_max_ and pathological features of primary tumor has been studied, its prognostic role was not confirmed [18-21]. There is limited evidence on impact of SUV_max_ in early-stage cervical cancer patients treated by surgery [19,20,22]. The present study intends to investigate the predictive role of SUV_max_ measured on preoperative ^18^F-FDG PET/CT for risk stratification of early-stage cervical cancer patients treated by surgery. Primary objective of the study was to assess association between SUV_max_ and recurrence rate or disease-free survival (DFS) or overall survival (OS), secondary objective was to assess the association between SUV_max_ and adverse clinicopathological parameters.

## Materials and methods

After approval from the institutional ethics committee (Roc.No.AS/11/IEC/SVIMS/2017, vide IEC No.1462) and informed consent, this prospective study was conducted between June 2018 to June 2019 in the Department of Surgical Oncology, Sri Venkateswara Institute of Medical Sciences, Tirupati, India. A total of 47 biopsy proven early-stage cervical cancer patients (FIGO stage I to IIA) aged between 18 to 80 years who underwent whole body ^18^F-FDG PET/CT followed by surgery (radical hysterectomy) were included. After surgery all pathological risk factors were evaluated and reported by experienced pathologist. Patients with pathological risk factors for recurrence received adjuvant treatment postoperatively. All patients were followed every three months during first two years, then every six months for subsequent two years. Information regarding age; FIGO clinical stage [23]; and clinicopathological features like tumor size, histology type, depth of invasion, LVSI, lymph node metastasis, parametrial involvement and oncological outcomes like recurrence rate, DFS and OS were collected and compared with SUV_max_ which was measured by ^18^F-FDG PET/CT.

Operational definitions: 1) Recurrence - Cancer recurrence was defined as when cancer was found in a patient after completion of treatment and a period in remission had passed; 2) DFS - It was defined as the time from end of treatment to recurrence of tumor or death related to cancer; 3) OS - It referred to the total duration of living time from the end of treatment to death due to any other cause.

Statistical analysis

All data was entered into Microsoft excel sheet. Statistical analysis was done by using SPSS software version 2021.Clinicopathological risk factors and the prognostic data were analyzed for association with the SUV_max_. Cut-off values of the SUV_max_ were determined by the receiver operating characteristic (ROC) curves. Study participants were divided into two groups. Group 0 with SUV_max_ below the cut off value (<7.65) and Group 1 with SUV_max_ above the cut off value (>7.65). Two-sample T test was used to compare the median SUV values in the different subgroups. DFS was calculated using the Kaplan-Meier method. The Cox proportional-hazards model was used for the multivariate analyses. Variables shown to be significant (P<0.05) in the univariate analysis were selected for the Cox model. A P-value of less than 0.05 was considered as significant.

## Results

The mean age of the study population was 48.38 ± 11.03 years. Of the patients, 10.6%, 23.4%, 25.5%, 36.2% and 4.3% were at FIGO (2009) stages IA, IB1, IB2, IIA1 and IIA2, respectively. Of the 47 patients, squamous cell carcinoma (SCC) was noted in 34 cases. The mean size of the tumor was 3.21 ± 1.71cm. Median SUV_max_ of the tumor was 11.80 (3.3-40). Lymph node involvement was seen in three cases and parametrium involvement was seen in three cases. The clinicopathological characteristics of study population were shown in Table 1.

**Table 1:** Clinicopathological characteristics of study population FIGO: International Federation of Gynecology and Obstetrics; SCC: Squamous cell carcinoma; AD: Adenocarcinoma; ASD: Adenosquamous

Characteristic	Number	Percentage
Total no. of patients	47	
Mean age at diagnosis	48.38 ± 11.03	
Initial FIGO stage		
IA1	5	10.6%
IB1	11	23.4%
IB2	12	25.5%
IIA1	17	36.2%
IIA2	2	4.3%
Histopathology		
SCC	34	72.3%
AD	3	6.4%
Small cell non-keratinizing SCC	2	4.3%
Large cell non-keratinizing SCC	4	8.5%
ASD	2	4.3%
Neuroendocrine carcinoma	2	4.3%
Mean size of the tumor	3.21 ± 1.71	
Median SUV_max_ (Min–Max)	11.80 (3.3–40.0)	
Pelvic lymph node involvement positive	3	6.4%
Median follow-up period in years (Min–Max)	2.0 (0.0–4.0)	
Mean follow-up period (years)	2.09 (1.44)	
Recurrence	7	14.9%
Site of recurrence		
Local	3	6.4%
Systemic	4	8.5%
Death related to cervical cancer	5	10.6%
Parametrium	3	6.4%

Association between SUV_max_ and clinicopathological parameters

Association between the SUV_max_ and clinicopathological factors is shown in Table 2. Significant difference in SUV_max_ was observed among the FIGO stage groups (P= 0.015). The mean SUV_max_ was significantly higher in patients with large tumor size (≥4 cm) compared to patients with tumor size less than 4 cm (P= 0.01). There was no significant difference in SUV_max_ between patient with positive pelvic nodes and negative pelvic nodes (P=0.639). The SUV_max_ of the tumor showing presence and absence of lymph vascular invasion was 12.95 and 10.35, respectively (P=0.5). The median SUV_max_ of tumors with depth of invasion ≥50% was almost thrice that of tumors with depth of invasion <50% (P=0.003).

**Table 2:** Association between SUVmax and clinico-pathological parameters SUV: Standardized uptake value

Risk factor for recurrence	SUV_max_	P-value
Pelvic lymph-node metastasis		0.639
Positive	10.36 ± 6.95	
Negative	12.28 ± 6.95	
Pathologic tumor size		0.001
<4cm	9.90 ± 5.00	
≥4cm	16.54 ± 7.64	
FIGO clinical stage		0.015
IA1	7.06 ± 3.48	
IB1	8.31 ± 5.33	
IB2	12.48 ± 4.48	
IIA1	15.80 ± 7.86	
IIA2	13.25 ± 5.16	
Lymphovascular invasion		
Present	Median SUV 12.95	0.5
Absent	10.35	
Depth of cervical stromal invasion		
<50%	Median SUV 4.7	0.003
>50%	13	

Correlation between SUV_max_ and recurrence rate

Correlation between SUV_max_ and recurrence rate is shown in Table 3. Recurrence rate in patients with SUV_max_ <7.65 and SUV_max_ >7.65 were 8.3% and 17.1%, respectively (P=0.65).

**Table 3:** Correlation between SUVmax and recurrence rate SUV: Standardized uptake value

SUV_max _cutoff	Recurrence rate	P value
<7.65	8.3%	0.65
>7.65	17.1%	

Correlation between SUV_max_ and DFS

**Figure 1:**
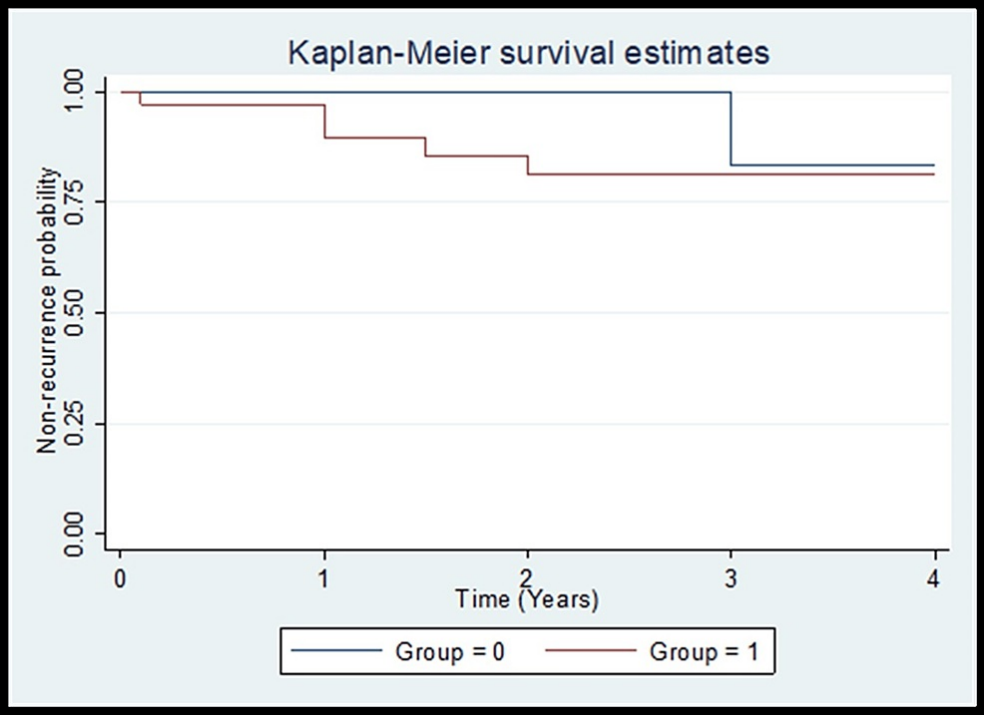
DFS in two groups with lower and higher SUVmax (cut-off value 7.65) Figure1: Group 0 indicates SUV_max_ < 7.65 and Group 1 indicates SUV_max_ >7.65. The difference between the two groups was statistically not significant (P=0.3, Log-rank test) SUV: Standardized uptake value; DFS: Disease-free survival

Correlation between SUV_max_ and DFS is shown in Figure 1. There was no difference in DFS between two groups (Group 0 with SUV_max_ <7.65 and Group 1 with SUV_max_ >7.65).

Correlation between SUV_max_ and OS

**Figure 2:**
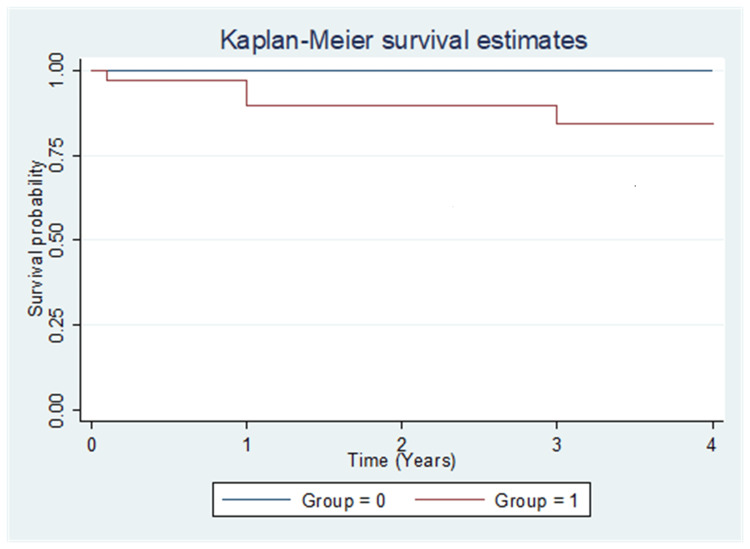
OS between two groups with low and high SUVmax (cut-off value 7.65) Figure 2: Group 0 indicates SUV_max_ < 7.65 and group 1 indicates SUV_max _>7.65. The difference between the two groups was statistically not significant (p=0.23, Log-rank test). SUV: Standardized uptake value; OS: Overall survival

Correlation between SUV_max_ and OS is shown in Figure 2. There was no difference in OS between two groups (Group 0 with SUV_max _<7.65 and Group 1with SUV_max_ >7.65; P=0.23).

Association of tumor size and recurrence

Association between tumor size and recurrence is shown in Table 4. Patients with tumor size >4cm had 2.1 times high probability of recurrence compared to tumor size <4cm, though not statistically significant (P=0.37).

**Table 4:** Hazard ratio for recurrence between the groups with tumor size <4cm and >4cm

Variables	Hazard ratio	95% CI	p-value
Tumor size <4cm	Reference		
Tumor size >=4cm	2.1	0.4 to 10.3	0.37

Association between tumor size and mortality

Association between tumor size and mortality is shown in Table 5. Patients with tumor size >4cm had 5.9 times more probability of mortality compared to tumor size <4cm (P=0.09).

**Table 5:** Hazard ratio for mortality between the groups with tumor size <4cm and >4cm

Variables	Hazard ratio	95% CI	p-value
Tumor size <4cm	Reference		
Tumor size >=4cm	5.9	0.6 to 57.1	0.09

## Discussion

There were conflicting results regarding predictive and prognostic role of SUV_max_ in cervical cancer patients (FIGO stage I-IV) treated by surgery, radiotherapy or palliative treatment [18,19]. These different results may be due to treatment bias as disease stages and treatment modalities were different. In early stage (IA-IIA) cervical cancer treated exclusively with surgery, there have been controversial studies on the role of SUV_max_, Lee et al. showed impaired DFS was correlated with high SUV_max_, while Crivellaro et al. showed increased recurrence was not associated with SUV_max_[19,21]. To clarify the predictive value of SUV_max_ the present study focused on FIGO stage IA -IIA who were only treated by surgery as primary modality. In present study, there was statistically significant difference between median SUV_max_ and FIGO stages, lower and higher stage tumor had lower and higher SUV_max_ respectively. Present study results are in concordance with study by Chung et al. that reported that higher FIGO stages are associated with high SUV_max_ (P =0.01) [22]. In contrast, Yu et al. reported that no statistical significance between groups with stage IB and IIA diseases in relation to SUV_max _(P > .05) [24]. In a study done by Yagi et al., SUV_max_ of the primary tumor on preoperative ^18^F-FDG-PET/CT was a prognostic indicator in patients with stage IA2 to IIB cervical cancer treated with radical hysterectomy [25].

Present study showed that a high SUV_max_ of primary tumor was significantly correlated with presence of conventional adverse clinicopathological risk factors such as tumor size, depth of cervical stromal invasion. In the present study, the median SUV_max_ of tumor <4cm and > 4cm was 9.7 and 13.6, respectively, which was comparable with study done by Xu et al., in which <4cm tumor SUV_max _was 9.77 and tumor > 4cm the SUV_max_ was 14.86 [26]. In present study and study done by Xu et al., there was statistically significant difference between the two groups, which means higher SUV_max_ correlates with large size tumor. In the present study, the median SUV of tumor in patients with cervical stromal depth of invasion>50% was 13, which was comparable with studies done by Xu et al. (12.44) and Zhang et al. [26,27]. Further, in these studies and the present study, there was statistically significant difference in SUV_max _between the two groups. Cut-off values of SUV_max_ for predicting lymph node metastasis was 6.03, cut-off for OS and DFS were 7.36 and 5.59, respectively, in all stages (IA-IIA). Consistent with present results, the study by Yun et al. showed that the cut off value of SUV_max_ >6 was predictive of DFS in stage IA-IIA [28]. In contrast, Lee et al. reported higher cut-off value (SUV_max_>13.4), which was predictive disease recurrence in stage IB1-IIA [19]. The study by Kidd et al. observed three subgroups according to the SUV_max_ cut off values low (<5.2), middle (5.2-13.3), and high (>13.3) [18]. Among these studies the cut-off values of SUV_max_ are different it can be due to ^18^F-FDG PET/CT settings, image analysis, condition of patient, and stage of disease.

In the present study, we did not find any significant differences in recurrence rate, DFS and OS among the two groups group with SUV_max_ <7.65 and SUV_max_ >7.65. Our findings are in concordance with study done by Crivellaro et al. [21]. In contrast, our findings are not in concordance with study done by Lee et al., who reported that in early-stage cervical cancer, tumors with high SUV_max_ (≥13.4) are at increased risk of recurrence [19].

In this study, the hazard ratio for mortality was 5.9 times higher in tumors >4cm compared to tumors <4cm (P=0.09), which is in concordance with study done by Wagner et al. that reported that with inclusion of size >4 cm for stage IIA cancers in new FIGO staging system for cervical cancer, it was better correlated with survival and overall prognosis [29]. Also, a study done by Kyung et al. reported that tumor size (4 vs 4-6 cm, P=.0371; and 4 vs >6 cm, P=.0024) was an independent predictive factor for the prognosis of stage II to IV cervical cancer [30].

Limitations

Other parameters of ^18^F-FDG PET/CT like metabolic tumor volume (MTV) and tumor lysis glycolysis (TLG) were not used along with SUV_max_ for prognostication in this study. Small sample size, variation in calculation of SUV_max_, histopathologic heterogeneity, and inclusion of stage IA patients in the study were some of the limitations observed in our study. Further studies using multi metabolic parameters of ^18^F-FDG PET/CT, including SUV_max_, SUV_mean_, SUV_peak_, MTV, and TLG are needed.

## Conclusions

SUV_max_ on preoperative whole body ^18^F-FDG PET/CT can be used to differentiate between stage I and II cancer and to predict unfavorable clinicopathological features in FIGO stage IA-IIA patients who have undergone radical hysterectomy. These findings suggest that the SUV_max_ of the primary tumour may be a promising marker for risk stratification in surgically treated, early-stage invasive cervical cancer patients. The present study did not find any difference in long term oncological outcomes between the groups; however, it showed higher hazard of recurrence and mortality in patients with tumor size > 4cm, which in turn correlated with higher SUV_max_. Future studies with large sample size and inclusion of other ^18^F-FDG PET/CT parameters along with SUV_max_ may throw light on their prognostic significance and individualizing treatment in early stage (IA2-IIA2) cervical cancer patients undergoing radical hysterectomy.

## References

[1] Bray F, Ferlay J, Soerjomataram I, Siegel RL, Torre LA, Jemal A (2018). Global cancer statistics 2018: GLOBOCAN estimates of incidence and mortality worldwide for 36 cancers in 185 countries. CA Cancer J Clin.

[2] Monk BJ, Tewari KS, Koh WJ (2007). Multimodality therapy for locally advanced cervical carcinoma: state of the art and future directions. J Clin Oncol.

[3] Yamagami W, Aoki D (2015). Annual report of the Committee on Gynecologic Oncology, the Japan Society of Obstetrics and Gynecology. J Obstet Gynaecol Res.

[4] Takekuma M, Kasamatsu Y, Kado N (2015). Reconsideration of postoperative concurrent chemoradiotherapy with fluorouracil and cisplatin for uterine cervical cancer. J Obstet Gynaecol Res.

[5] Takeda N, Sakuragi N, Takeda M (2002). Multivariate analysis of histopathologic prognostic factors for invasive cervical cancer treated with radical hysterectomy and systematic retroperitoneal lymphadenectomy. Acta Obstet Gynecol Scand.

[6] Singh N, Arif S (2004). Histopathologic parameters of prognosis in cervical cancer-a review. Int J Gynecol Cancer.

[7] Kasamatsu T, Onda T, Sawada M, Kato T, Ikeda S, Sasajima Y, Tsuda H (2009). Radical hysterectomy for FIGO stage I-IIB adenocarcinoma of the uterine cervix. Br J Cancer.

[8] Mabuchi Y, Yahata T, Kobayashi A (2015). Clinicopathologic factors of cervical adenocarcinoma stages IB to IIB. Int J Gynecol Cancer.

[9] Rosa DD, Medeiros LR, Edelweiss MI (2012). Adjuvant platinum-based chemotherapy for early stage cervical cancer. Cochrane Database Syst Rev.

[10] Ryu HS, Chun M, Chang KH, Chang HJ, Lee JP (2005). Postoperative adjuvant concurrent chemoradiotherapy improves survival rates for high-risk, early stage cervical cancer patients. Gynecol Oncol.

[11] Katal A, Al-Ibraheem A, Abuhijla F, Abdlkadir A, Eibschutz L, Gholamrezanezhad A (2023). Correlative imaging of the female reproductive system. Radiology-Nuclear Medicine Diagnostic Imaging.

[12] Tanizaki Y, Kobayashi A, Shiro M (2014). Diagnostic value of preoperative SUVmax on FDG-PET/CT for the detection of ovarian cancer. Int J Gynecol Cancer.

[13] Kitajima K, Kita M, Suzuki K, Senda M, Nakamoto Y, Sugimura K (2012). Prognostic significance of SUVmax (maximum standardized uptake value) measured by [¹⁸F]FDG PET/CT in endometrial cancer. Eur J Nucl Med Mol Imaging.

[14] Antonsen SL, Loft A, Fisker R (2013). SUVmax of 18FDG PET/CT as a predictor of high-risk endometrial cancer patients. Gynecol Oncol.

[15] Nakamura K, Hongo A, Kodama J, Hiramatsu Y (2011). The measurement of SUVmax of the primary tumor is predictive of prognosis for patients with endometrial cancer. Gynecol Oncol.

[16] Al-Ibraheem A, AlSharif A, Abu-Hijlih R, Jaradat I, Mansour A (2019). Clinical impact of (18)F-FDG PET/CT on the management of gynecologic cancers: one center experience. Asia Ocean J Nucl Med Biol.

[17] Kidd EA, Siegel BA, Dehdashti F, Rader JS, Mutch DG, Powell MA, Grigsby PW (2010). Lymph node staging by positron emission tomography in cervical cancer: relationship to prognosis. J Clin Oncol.

[18] Kidd EA, Siegel BA, Dehdashti F, Grigsby PW (2007). The standardized uptake value for F-18 fluorodeoxyglucose is a sensitive predictive biomarker for cervical cancer treatment response and survival. Cancer.

[19] Lee YY, Choi CH, Kim CJ (2009). The prognostic significance of the SUVmax (maximum standardized uptake value for F-18 fluorodeoxyglucose) of the cervical tumor in PET imaging for early cervical cancer: preliminary results. Gynecol Oncol.

[20] Pinho DF, King B, Xi Y, Albuquerque K, Lea J, Subramaniam RM (2020). Value of intratumoral metabolic heterogeneity and quantitative (18)F-FDG PET/CT parameters in predicting prognosis for patients with cervical cancer. AJR Am J Roentgenol.

[21] Crivellaro C, Signorelli M, Guerra L (2012). 18F-FDG PET/CT can predict nodal metastases but not recurrence in early stage uterine cervical cancer. Gynecol Oncol.

[22] Chung HH, Nam BH, Kim JW (2010). Preoperative [18F]FDG PET/CT maximum standardized uptake value predicts recurrence of uterine cervical cancer. Eur J Nucl Med Mol Imaging.

[23] Pecorelli S, Zigliani L, Odicino F (2009). Revised FIGO staging for carcinoma of the cervix. Int J Gynaecol Obstet.

[24] Yu L, Jia C, Wang X, Lu P, Tian M, Wang W, Lou G (2011). Evaluation of ¹⁸F-FDG PET/CT in early-stage cervical carcinoma. Am J Med Sci.

[25] Yagi S, Yahata T, Mabuchi Y (2016). Primary tumor SUV(max) on preoperative FDG-PET/CT is a prognostic indicator in stage IA2-IIB cervical cancer patients treated with radical hysterectomy. Mol Clin Oncol.

[26] Xu W, Yu S, Xin J, Guo Q (2016). Relationship of 18F-FDG PET/CT metabolic, clinical and pathological characteristics of primary squamous cell carcinoma of the cervix. J Investig Med.

[27] Zhang L, Sun H, Du S, Xu W, Xin J, Guo Q (2018). Evaluation of 18F-FDG PET/CT parameters for reflection of aggressiveness and prediction of prognosis in early-stage cervical cancer. Nucl Med Commun.

[28] Yun MS, Kim SJ, Pak K, Lee CH (2015). Additional prognostic value of SUVmax measured by F-18 FDG PET/CT over biological marker expressions in surgically resected cervical cancer patients. Oncol Res Treat.

[29] Wagner AE, Pappas L, Ghia AJ, Gaffney DK (2013). Impact of tumor size on survival in cancer of the cervix and validation of stage IIA1 and IIA2 subdivisions. Gynecol Oncol.

[30] Kyung MS, Kim HB, Seoung JY (2015). Tumor size and lymph node status determined by imaging are reliable factors for predicting advanced cervical cancer prognosis. Oncol Lett.

